# Types of gambling: finnish gambling narratives under the lens of systems theory

**DOI:** 10.3389/fsoc.2023.1199474

**Published:** 2023-08-02

**Authors:** Michael Egerer

**Affiliations:** Centre for Research on Addiction, Control, and Governance (CEACG), University of Helsinki, Helsinki, Finland

**Keywords:** gambling, systems theory, narratives, typology, Finland

## Abstract

There seems to be no shortage of gambling and problem gambling typologies. At a closer look, however, previous research identified types of problem gamblers and not of problem gambling. While correct typologies of gamblers are important for developing treatment, they are less useful for harm prevention. The current study uses a system theoretical approach to investigate gambling communication in order to develop a genuine typology of gambling. Snowball sampling of Finnish gamblers resulted in 56 participants, who wrote 48 narratives about their ordinary gambling, 43 narratives about their most remarkable gambling event, and 28 about their worst gambling experience. The approach is informed by systems theory: communication on gambling is understood as a result of the reduction of contingency. Rather than focusing on the meaning of gambling or why people gamble, the analysis investigates what is included and what is excluded to make gambling discussable, i.e., the contextures of gambling. Economic and family/intimate contexture were the most prominent. The latter appeared most often in the most memorable gambling experiences. The economic contexture was more common in narrating ordinary and worst gambling situations. In all, four types of gambling could be identified: genuine monetary gambling, resonating monetary gambling, commensal gambling, and liminal gambling. When comparing the previously identified types of gamblers with the types of gambling discovered in the present study, it becomes obvious that a shift from the gamblers, their background, their personality, and their motives to the gambling activity provides novel insights. The constant appearance of the familial/intimate dimension in the narratives indicates that, beside the financial harms, societal harms also need to be treated as a category of harm in its own right, not just as a consequence of personality disorders, psychological distress, or social deprivation.

## Introduction

The scientific discourse understands problem gambling mainly in terms of its psychological symptomatology and the individual's cognitive characteristics (e.g., Martignoni-Hutin, 2005; Bjerg, 2010; Hirschovits-Gerz et al., 2012). The common screening instruments for problematic gambling identify the disorder by the cognitive malfunctions and the adverse consequences of the gambling activity (e.g., SOGS, PGSI, DSM-V, or ICD-10). Typologies of problem gambling are based likewise on the individuals and their psycho-social characteristics.

This approach, however, neglects the changeability of one's personality in the social context, and disregards the variability of the gambling activity itself (e.g., Bjerg, 2010; Reith and Dobbie, 2011; Matilainen and Raento, 2014; Egerer and Marionneau, 2015; Kristiansen and Trabjerg, 2017). In fact, previous research identified types of problem gamblers and not types of problem gambling. While typologies of gamblers are important for developing treatment, these typologies are less useful for harm prevention. Prevention efforts need to account for types of games, environments, and supply factors to identify risky gambling trajectories and to promote low-risk gaming and gambling.

Before problematic gambling was included in the diagnostic manuals (i.e., DSM-III or ICD-9), Moran (1970) established a typology of problem gamblers. He identified a subcultural variety, a neurotic variety, an impulsive variety, a psychopathic variety, and a symptomatic variety. Although Moran calls these varieties of problem gambling, they are, in fact, varieties of problem gamblers with distinct aetiologies of the pathology. The wide-spread typology of problem gambling by Blaszczynski and Nower (2002) presents three pathways into problem gambling. The three subtypes of gamblers are “Behaviourally conditioned,” “Emotionally vulnerable,” and “Antisocial impulsivist problem gamblers.” While these types are more elaborated on than the early attempts to classify problem gambling, the Blaszczynski and Nower typology concerns gamblers' characteristics and their ways into problem gambling.

Sociological typologies are more sensitive to the social setting and also examine recreational forms of gambling. Fisher (1993) identified five types of gamblers in young slot-machine players by putting special emphasis on the social relations between the players as well as their motivations to gamble. These are the Arcade Kings and their Apprentices, Machine Beaters, Escape Artists, Action Seekers, and Rent-a-Spacers. In an ethnographic study of German gaming halls, Reichertz et al. (2010) distinguished between the perspectives of those who categorise problem gamblers: researchers, game providers, and gamblers themselves. Whereas, game providers categorise between gamblers and real gamblers–depending on the economic value of and possible trouble with the gamblers–gamblers themselves distinguish between novice, competent, and problem gamblers. Gamblers tend to place themselves in the middle, i.e., to consider themselves as competent gamblers. The researchers, as others before them, looked at the motives of gamblers in order to categorise them and found nine types of gamblers. There included “thrill hunters,” “money hunters,” or “investigators of fate.” For all types of gamblers, gambling involves working on the identity.

While not genuine typologies of gambling, some research studied the games involved in gambling. Bjerg (2010) studied poker as a social game of skill and elaborated the specific pathway into problem poker. In comparison to bank games of chance, loss of money, control, and irrationality have a different position in the creation of problem poker. Distinguishing games of chance from games of skill by using Roger Caillois' (1961) classification of play, Young and Stevens (2009) and Stevens and Young (2010) relate these types of games to gamblers' biographical data. They also cheque for a correlation between problem gambling and the type of game. The authors nevertheless do not inquire into changes in the gambling activity, but rather understand the type of game as a stable activity, depending on chance or skill. Such a distinction, however, is problematic, as it neglects gamblers' own view on their game and dismisses their gambling strategies simply as irrational (Oldman, 1974). Egerer and Marionneau (2015) looked at the corruption of play, or changes in the gambling activity itself. They also referred to Caillois' (1961) classic terminology but put the focus on the characteristics of play and their “corruption,” and not on Caillois' classification of forms of play. With this framework they analysed general practitioners' understanding of the border between recreational and problem gambling. Depending on the cultural context (Finland, France, and Germany), GPs indeed discuss different transition points from recreational to problem gambling and describe how the play becomes corrupted in its different characteristics.

Matching this line of research concerning the gambling activity itself, and in order to focus on developing a typology of gambling (rather than of gamblers), the present study harness a theoretical approach where gambling can be analytically insulated from human individuals: the system theoretical approach. The system theoretical approach is particularly suitable for tilting the lens away from the human individual, the gambler, and towards gambling itself (Egerer et al., 2020). In systems theory social systems are autopoietic, i.e., self-sustained and involve communication (Luhmann, 1984). Human individuals might be necessary as the hardware of communication but are not part of the social systems themselves. From a Luhmannian perspective, humans instead are the environment of the social systems which consist solely of communication.

Communication is troubled by the matter of double contingency. Contingent refers to something that is as it is, but could have been also different (Luhmann, 1984; p. 152). The environment of a social system and the matter of contingency can be well described with the metaphor of indistinguishable background noise (see for instance Stäheli, 2003 on the role of noise in the origins of the stock exchange). Adjusting to such indistinct noise, i.e., filtering according to the needs and logic of the listening or observing system, is the way to make sense and receive information (Wiener, 1954). In all communication, double contingency comes into play. Double contingency is a matter in communication as both the sender and the receiver need to take into account alternate possible intentions and understandings, but in addition also alter one's own processing and anticipation of the ego's world (Luhmann, 1984). Both experience double contingency and, as a result of the innate uncertainty of the situation, any word or sentence generates order (ibid., p. 154). An order can be anticipated and enable communication. The communication about gambling that will be under scrutiny in this study is 119 written accounts of particularly negative or positive gambling experiences, as well as of instances of routine gambling.

In the following, I will describe the materials and methods used in developing genuine gambling types as well as the system theoretical framework of the analysis in more detail. After this, I will report on the result, concluding with a discussion on the implications of my findings.

## Materials and methods

### Method and context

This study uses elicited written data (Matilainen and Raento, 2014): individuals' written stories or narratives about their gambling. Participation in the study included using a link to an Internet form. The anonymous Internet form gave a description of the study and short instructions about the three categories of narratives we were looking for (see Appendix): ordinary gambling experience, most memorable gambling experience, and the worst gambling memory. Asking about these three different kinds of gambling narratives was inspired by Zinberg's (1984) ground-breaking work on the set and setting of drug use, with the aim to learn about usage as well as the whole continuum of experiences. The participants of the present study did not need to write all three narratives asked for but could leave some categories blank. At the end of the form, the phone number of the problem gambling helpline was listed. It was also possible to send the narratives via an email attachment, though none made use of this option. In a separate form, the study participants could leave their postal address in order to receive the 20€ supermarket voucher we offered as a compensation for their time and efforts (Head, 2009). The data were collected during December 2017. The University of Helsinki Ethical review board in humanities and social and behavioural sciences has evaluated the study with a positive verdict (Statement 17/2017).

Finland is a country with very high gambling participation and per capita gambling losses (Salonen et al., 2020). The Finnish gambling culture has been characterised by the wide availability of EGMs in everyday spaces, such as petrol stations and supermarkets, and, until recently, a positive public opinion of the state-owned gambling monopoly, Veikkaus. The positive reputation has been greatly due to the visibility of supporting charitable causes with the gambling revenue (Matilainen, 2017). The data were collected at the beginning of a changing public perception of gambling and the move towards a more critical one (e.g., Egerer et al., 2018). The collected data did, however, show no signs of this criticism, probably due to the kind and character of the data and its collection.

### Data

The participants were recruited via a snowball sampling strategy. We informed potential participants about the study in a parallel focus-group study conducted with Finnish residents (Egerer et al., 2018), and asked the focus-group participants to spread the word (and share the link to the internet form) in their networks. The focus-group participants themselves had various gambling experiences, from gambling not at all towards heavy gamblers (ibid.). We did not pre-determine any level of gambling experiences as a prerequisite for the present study. In order to keep the participation as simple and short as possible, we refrained from asking about the participants' gambling expenses or any other background information beside age and gender. Taking the system theoretical angle (see the following section: Method of analysis), the focus was on the communication and how gambling becomes communicable, not on the person and their background. In total, 56 (33 women, 20 men, three other/did not want to say) people participated. We collected 48 narratives about participants' ordinary gambling, 43 narratives about their most memorable gambling experience, and 28 about their worst gambling experience. We did not pre-determine the length or style of the narratives. The narratives were of various length (from 7 to 549 words) and quality. In general, the narratives on the most memorable gambling experiences were longer and the worst gambling experiences shorter, though there were also exceptions. I excluded one narrative about the worst gambling experience from the analysis, as it constituted an unintelligible text fragment.

### Method of analysis

The analysis is based on a practical and pragmatic implementation of the systems theoretical toolbox (Vogd, 2011) rather than systems theory as a grand societal theory. A full discussion of systems theory would thus go beyond the scope of this article and the following elaboration will emphasise the presentation of the main concepts informing this study as well as the methodological aspects (see Egerer et al., 2020 for a more detailed description of systems theory in relation to the study of gambling in various dimensions).

Social systems, like the economy or politics, consist of communication, reducing the environmental complexity and anticipating connectable further communication (Luhmann, 1984). Systems never merge. For any one system, another system is merely part of its environment. When looking at the societal reality, one cannot avoid recognising systems as having an impact on other systems. A system needs to process the complexity produced by another system and, for example, an economic transaction is impacted by the legal environment. The economic systems observe economic logic and are impacted by what has happened in the legal system. Vogd (2011) uses the metaphor of resonance to explain how a system reacts to the produced complexity of another system. The two systems remain clearly distinct but adjust their ‘wavelength' on what they receive from the outside. The concept of resonance has been introduced to the English reader in *Ecological Communication* (Luhmann, 1989).

In the functional differentiated societies of today, the function systems have a special place. Function systems are sub-systems of the overall social system, which process a higher degree of environmental complexity as non-functional systems. What qualifies as a function system remains a matter of debate but one can add with confidence the economy, science, the legal, and the political systems (Roth and Schütz, 2015). An important characteristic of function systems–and what makes them so efficient in reducing complexity–is the generalised media of communication. A generalised medium of communication is a standardised form of communication that considerably raises the likelihood of successful communication of the system. For the economic system, for example, this generalised medium is money. While the economy can obviously function without money, the economic system would be much more unstable and prone to halt, as each economic affair would be dependent on the contingent situation in every economic communication anew. This is important to keep in mind in the scope of the present study, as the same applies for gambling, which can happen without money, but is profoundly facilitated by the existence of a differentiated function system of economy (Binde, 2005).

In a similar way to understanding social systems and their communication as reducing complexity, one can look at the communication happening in the research data (Nassehi and Saake, 2002), i.e., here the narratives about ordinary, memorable, and worst gambling. The analysis informed by systems theory tries to identify what is included and what is excluded to talk about gambling. While there are an infinite number of possibilities of what to say (include or exclude), the progression of the narrations themselves gives an indication on how the complexity is reduced in the data. Simply said, each sentence follows the other and, by so doing, shows what is included and in which order. In addition, comparing the narratives allows identifying what one narrator leaves unsaid and the other deems necessary to add (e.g., the space where the gambling happened, or the amount staked).

In comparison to many strategies for analysing qualitative data, the main question under a system theoretical framework is not what the narrator means, what gambling means, or what underlying meaning could be found in the narrative, but how the narrator makes it possible to talk about gambling (Nassehi and Saake, 2002). How is the narrator able to establish and ensure communication on gambling? To understand what lies behind the data, the analysis looks for contextures. A contexture is a frame or logic used to make something relatable and understandable (Vogd, 2011). It constitutes a distinct way or angle (like a system) of observing the reality (Larsson et al., 2020). Contextures can, for example, make gambling communicable by placing it in an economic frame (e.g., talking about the size of the stake) or a familial/intimate frame (e.g., mentioning the presence of one's spouse while gambling). Narrations are usually built-up of various and alternating contextures, i.e., polycontextural structures, which resonate inside each other. “Such networks are dependent on the observer who replaces any linear causality assumed in actor-based analytical frameworks.” (Egerer et al., 2020, p. 18). Contextures are not contexts. Whereas, contexts are the point of reference, stating what is happening (when and where), contextures link the context with the standpoint of the observer (cf. Knoblauch, 2021).

Practically, the analysis was conducted in two stages. In the first stage, the data were organised into broad categories (Deterding and Waters, 2021) by identifying the most pronounced contexture of each of the narratives. In the second step, the narratives were revisited in order to understand possible polycontextural networks (Vogd, 2011) of the narratives and, through this, develop the various types of gambling present in the data. The original Finnish narratives served as the data for the analysis. The example quotations in the following results section were translated by the author. They aim at exemplifying the main characteristics of the different types of gambling. Yet, as the types of gambling are meant as abstractions from reality, serving as heuristic tools, these never match the real narratives exactly (Swedberg, 2018).

## Results

**Figure 1:**
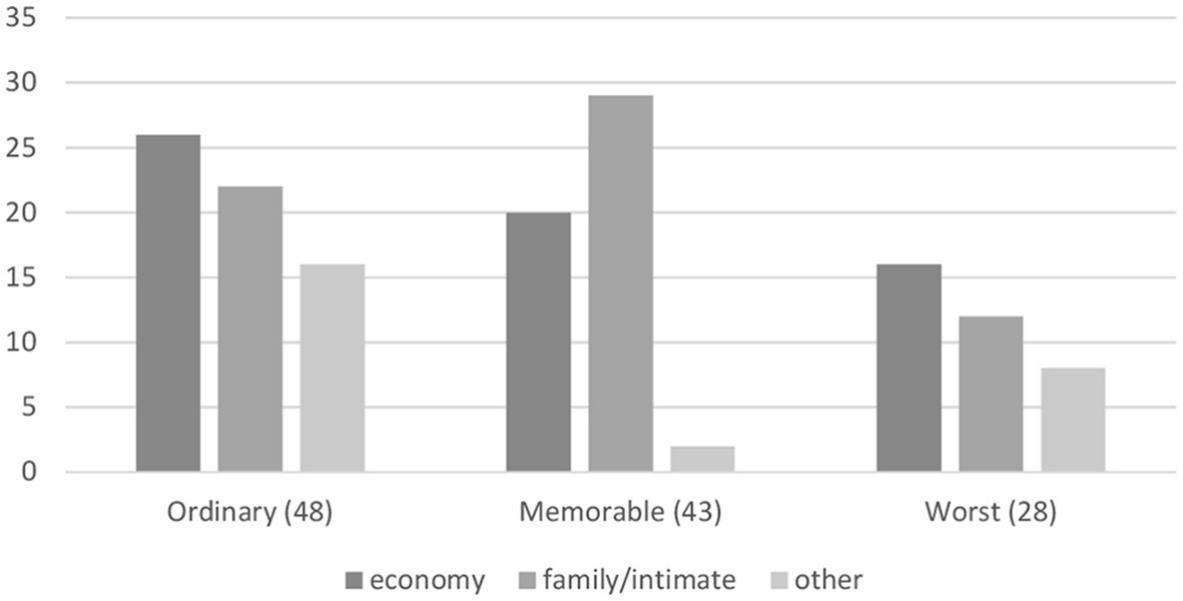
Dominant contextures.

Overall, an economic and a family/intimate contexture were the main forms of recounting one's gambling (see Figure 1). The economic frame was most prominent in the ordinary as well as in the worst gambling experience narratives. A family or intimate contexture, on the other hand, was the most common for the most memorable gambling events reported. There were also instances of other contextures, such as a medical one. Nevertheless, and despite the medicalisation of problem gambling (e.g., Castellani, 2000; Bernhard, 2007; Ferentzy and Turner, 2013), the medical contexture remained marginal in comparison to the economic and family/intimate contexture, even in the category of worst gambling experiences.

### The economic types of gambling: genuine monetary gambling and resonating monetary gambling

The first type of economic gambling could be considered the epitome of gambling itself: winning and/or losing of money. Gambling is a matter of economic transaction and money in its pure sense. This type consequently will be named “*genuine monetary gambling.”* In Simmel's Philosophy of money (Simmel, 1900), money is a neutral means of exchange. Because it has no assigned use value on its own, money holds countless possibilities (of real possession). Money itself does not assume any particular transaction. The narratives underlying the type of genuine monetary gambling were largely short and straightforward stories, with little contextualisation. The economic contextural structure remains rather simple, interchanging only rarely with other contextures.

“*It happened about a week ago. I put 2 euros in the gambling machine at the kiosk at the train station. I was on my own. I played with one-euro stakes a typical poker game and lost. The experience was emotionally quite neutral and the outcome [of the game] to be expected. After the game I quit the place – before playing I did buy a coffee at the kiosk.” (Man, 28 years, ordinary gambling)*

As the example above shows, after a temporal contextualisation the focus lies on the use of two euros for gambling and its loss. Only the account of gambling breaks the otherwise mono-contextural structure. The gambling narrative even ends with another economic transaction (buying a coffee). The economic contextural structures remain rather simple, changing only rarely to other contextures. The narrators anticipate that it is clear what they are talking about and the few words they use are sufficient for continuous communication (cf. Egerer et al., 2020). This applies particularly to the majority of narrations of worst gambling experiences where, often and maybe not surprisingly, losses are also faster and/or more significant.

“*Sometimes, greater sums [of money] were lost in gambling than initially planned, and of course this has been frustrating at times.” (Woman, 27 years, worst gambling)*

In the example above, the narration might switch from an economic contexture to a psychic dimension, yet it is taken as self-evident that the change in the economic contexture resonates in the psyche. While the memorable gambling narratives constituted usually richer, polyconextural networks, *genuine monetary gambling* was also occasionally reported on in this category.

“*It felt good when I won 22*>€ *in the lottery. I drank the money [used the money to buy alcohol].” (Man, 42 years, most memorable gambling)*

This very short narrative exemplifies well what the narrator anticipates as being necessary to communicate a positive form of gambling–very little in fact. The narrator could have added, for example, contextual information about the time as in the narrative above (or about the space as in the next narrative example) or given more details on the kind of drinking occasion he used the winnings for. Yet, winning money and the use of the winnings for some (kind of) luxury is expected to be enough information enabling the reader to understand and has the possibility to continue the communication about gambling. More often, however, the memorable gambling narratives tended to be lengthier, binding different contextures together.

“*[Hypermarket in city X]. My adult daughter said that I could play a few euros, if I give her 50*>€ *of the possible win. How surprised we were when I won almost 200*>€*! This way my daughter got the promised amount and we were happy for being on the winning side for once.” (Woman, 60 years, most memorable gambling)*

The economic contexture with monetary transactions still makes up a considerable part of this type of gambling, but the narrative also reports how the money transactions are interwoven with other contextures: in the case of the example quotation above that would be the familial relationship between mother and daughter. Referring to Zelizer (1997) and questioning Simmel's (1900) neutrality of money, Kinnunen et al. (2016) discuss the different meanings attached to the money as the stake for gambling. While nominally the same, gamblers consider and manage money differently depending on the origin of the money. The scene of the narrative is set by giving the information on the place of happening–a big supermarket in a Finnish city. In comparison to the previous narrative, the narrator deemed the spatial information to be important for the reader to understand what kind of gambling she engaged in. Here gambling is interwoven with one's groceries, being a re-occurring finding in previous research on Finnish gambling habits (e.g., Kinnunen et al., 2012; Egerer and Marionneau, 2019). Buying one's groceries can be understood as being part of the economic contexture. Yet, by mentioning her daughter, a familial/intimate contexture also comes into play: shopping is not only done for oneself, but also for the family. In addition, the two contextures are also interwoven in sharing the won money within the family. Hence, gambling here is a phenomenon intersecting the economic as well as the familial realm. In system theoretical terms, both contextures resonate within each other (Vogd, 2011), and this type of gambling will thus be called *resonating monetary gambling*. In this type of gambling, the economic contextures and the monetary exchange remain prominent as compared to the familial/intimate types of gambling described in the following section. The economy resonates in other contextures rather than the other way around.

Resonating monetary gambling is also a matter of ordinary gambling, where gambling together with one's spouse, parents, or friends has been a reoccurring narrative, but were the economic interaction remains the centre of the narrative. Worst gambling narratives with a prominence of an economic contexture, on the other hand, constituted largely genuine monetary gambling.

### The familial/intimate types of gambling: commensal and liminal gambling

Gambling is often a very lonely activity, with the gambler interacting only with the game itself, though it can be also a shared activity. Sharing is also a core dimension in the sociological study of food and the meal. As with food, money (i.e., the stake) can only be consumed by one person once, whereas the gambling experience can be shared. Simmel (1984) described the meal as an occasion where the food itself cannot be shared, instead it is exclusively reserved for one person (i.e., can only be eaten once). Yet, the shared table often using a similar set of dishes facilitates the experience of a common meal. Claude Fischler (2011) uses the concept of commensality to describe the bonding capacity of a shared meal. Shared gambling was also a recurring theme in the narratives, and this type of gambling shall be named *commensal gambling*.

“*My most common gambling experience is the weekly lottery line, which I set automatically at my Veikkaus [the Finnish gambling monopoly company] account. […] My partner and I were at a party, so* ***this weekly lottery line is a joint project****. [emphasis added] To some extent it has been surprising how much fuss this one euro weekly lottery line creates (“Did you check the lottery”, “Did we win something in the lottery” etc.); and one even manages to be disappointed when you are not winning anything, […]. Maybe the reason is also that if the line really won, it would be a pretty good story to tell there at the same party next year!” (Man, 44 years, ordinary gambling)*

In the narrative above, the weekly lottery is an activity tying not only the spouses together, but also binding them to the participants of the mentioned party. While the stake is spoken of briefly, the same sentence is largely embedded in the familial/intimate contexture, focusing on the “fuss,” or the interaction that gambling creates between the spouses. This category also contains several narratives on how gambling habits are inherited inside families “over generations” (Matilainen and Raento, 2014: p. 438). While we had not specifically asked participants to describe how they learned gambling, the respondents tended to fit their gambling learning experiences mainly into the category of ordinary gambling experiences. Gambling can strengthen the relationship between a parent and a child (ibid.), while re-occurring (here: weekly) gambling can maintain social relations between spouses. Yet, in a similar way, commensal gambling can create poor memories and disturb relations.

“*I was maybe 25 years old. I had never filled a sports betting slip when my aunt asked me to fill her slip. I filled the slip according to my aunt's advice. She wanted to check the slip before I bring it to the kiosk. My aunt got mad, as if I somehow marked the boxes wrongly. She got upset with me for filling the slip so messily, asking if I had never bet before. When I said that I had not, she did not believe me*. ***My aunt was*** ***angry with me for many weeks*** *[emphasis added] for not knowing how to do such a simple thing and even lied that I've never bet.” (Woman, 60 years, worst gambling)*

The narratives happen largely inside a familial/intimate contexture. While in the above example, the gambling narrative did not report on any form of problem gambling nor even gambling losses, it shows how gambling can bind people together in a negative way (also Borch, 2013; Salonen et al., 2014). Several worst gambling experiences did in fact also focus on how losses strained partnerships and families.

The prominence of the familial/intimate contexture became particular obvious in the following narrative which could easily be misread as an instance of problematic gambling, but which in fact is a report of a woman's most memorable gambling experience.

“*Once I was in love with a man and we went for pizza and beer. He withdrew 200*>€ *[from an ATM] and I was allowed to gamble the whole amount. I lost the whole sum in the end, but I could gamble to my heart's and soul's content.” (woman, 50 years, most memorable gambling)*

The experience refers to monetary value and economic transactions (i.e., 200€ and the loss of the full sum), but these are embedded in the familial/intimate contexture as a sign of love. Rather than understanding love simply as an emotion, one can also understand it as a code of communication (Luhmann, 1982). Love as a code entails blueprints of behaviour, making an otherwise unlikely communication happen–unlikely because the lovers as individuals need to relate towards each other in their entirety, without the possibility to observe the inside of the other (ibid.). One can only assume love based on the ‘output' (ibid., p. 28). This highly personalised form of communication (Morgner, 2014), is under continuous threat of failing. One cannot read the other's mind and is instead in need of, often small, indicators. Thus, the established forms of showing one's love. In the example narrative above, the indicator is gifting a considerable amount of money for gambling, an activity which was fulfilling to the narrator. This stresses the capacity of love as a code for communication, by contrasting the wasting of money with the ability to do so without a bad conscience. The narrator could have also framed the narrative economically by, for example, describing a negative reaction from her date due to the lost money, but instead she focuses on how her date's gift enabled her to enjoy gambling to the fullest. Whereas, the reciprocity has been established as one of the main functions of a gift (i.e., Marcel Mauss' gift theory), here the anticipated non-reciprocity in fact serves in communicating love.

The final type of gambling identified is *liminal gambling*. Liminality is a concept regularly used in anthropology. Liminality describes a transitory passage or situation void of the common rules, norms, or habits of society (e.g., Szakolczai, 2009; Thomasson, 2009). Applying a similar idea, already Huizinga (1939), sees play as a realm apart from ordinary life. In relation to gambling, Näre and Laähteenmaa (2017) talk about an increased and persistent liminality in our present capitalist society. Here, I understand liminal gambling once again more in the anthropological sense, looking at temporary forms of transitions and the separate islands from everyday conventions. Egerer and Marionneau (2019), coined the term gambling rausch in order to describe forms of gambling which are apart from everyday life. Yet, the gambling rausch also entails altered states of consciousness (ibid.), which are different from the type of liminal gambling described in the following. The narratives are often more mundane in character when compared to the gambling described in the earlier study (ibid.).

Traditional festivities throughout the year are instances of liminality and *Christmas* was a frequent theme in the narratives. The high frequency of mentions of Christmas is most likely due to the time of data collection (i.e., December) but, nevertheless, the underlying contextural network in these narratives follows a common structure independent of the specific festivity.

“*I received the scratch-ticket as a Christmas present, I won, I think, 5*>€ *from it. The shop clerk managed to persuade me to buy a new one. […] On our regular trip to a cottage in Lapland with friends for New Years Eve, […] I checked it out, out of boredom, on the 14-hour drive. I saw that I won something like 6*>€*. […] At the next gas station we stop, I thought, I am going to buy my coffee with it. To my surprise, the shop clerk there asked me if I want the rest in cash or on my account. I was like,…? What? […] The shop clerk told me I had won 60*>€*. I had to laugh a lot. […]* ***My friend behind me laughed and it was fun for everyone; also the shop*** ***clerk laughed*. ***[emphasis added] The winnings went into our joint cashbox for food [on the trip].” (woman, birth year not mentioned, most memorable gambling)*

In the narrative presented in the previous section, a gift (i.e., 200€) enabled carefree gambling; in the example above, on the other hand, the narrative is set in motion by the money won due to gambling as a gift (i.e., a scratch-ticket). In addition, the liminal type of gambling is also one that is communicated typically in a familial/intimate contexture, and which alternates with an economic contexture. Yet, the economic contexture remains a rather short backdrop in the narrative. The bonding element of gambling stands out and spreads beyond the immediate social circle of the gambler (“*also the shop clerk laughed”*) and the immediate gambling event. The scratch-cards as a gift bind the narrator to the donor, whereas the win binds the travel companions (“*our joint cashbox”*). The narrative reports on three regular but transitory events: Christmas, New Year's Eve, and the trip to the cottage in Lapland. Gambling becomes discussable in the context of these three events. Obviously, the narrator could have chosen to mention the scratch-card as a present, without mentioning the gift being a Christmas present. Likewise, the story would be understandable without knowing that it happened at the end of the year. Yet, the narrator anticipated these circumstances as important for the reader to fully understand her most memorable gambling experience.

Once again, gambling not only serves as a bonding, but also binding experience, and gambling as a gift can also be a matter of negative gambling experiences.

“*My worst gambling memory is related to the Christmas calendar scratch cards. I bought them regularly for a few years for my godsons, my own son and my husband. As far as I remember, there were only two wins in these twenty draws. […] But then it somehow also occurred to me that if one of them wins a large sum and the others do not. The bad feelings it would cause to the others. That not-even-teenage boys would share their win might be unrealistic. So, I decided to give up the scratch cards and get different types of calendars instead.” (Woman, 55 years, worst gambling experience)*

Placed foremost in a familial/intimate contexture, the randomness of gambling is a possible matter of concern, even in case of one, or more so, two wins. The issue in this narrative is not the winning or losing in economic terms–the exact amounts for example are not reported on, i.e., are not deemed important in talking about this worst gambling experience–but the proportionality and fairness of the wins between the family members and relatives. Gifts create social relations in assuming reciprocity, which is rare in the gambling field (Järvinen-Tassopoulos and Eräsaari, 2018). This puts gambling as a gift in a contradictory position. As demonstrated in the example narrative further above, assuming non-reciprocity can be an indicator of love; in the present example, however, the question is about equal gifts and thus establishing equal social relations. Other narratives on the worst gambling experiences also brought up the matter of (Christmas) gifts, but there the regrettable gambling experience was more due to the meaning attached to the money (Zelizer, 1997) and a consequential double feeling of loss (loss in economic but also personal terms), when having lost the money gambling.

The liminal type of gambling could logically not appear in the ordinary gambling narratives, but were present as most memorable, as well as worst gambling experiences. Beside the narratives about Christmas (presents), the second form of the liminal type of gambling reported on music festivals or–as the narrative example above does–holidays. These holidays and trips were mostly abroad. Most of these gambling narratives concerned gambling on the Baltic ferries connecting Finland with Estonia and Sweden. These ferries offer all kinds of entertainment, are known for heavy alcohol drinking and merry-making, and could be considered a prime example of liminality (for a discussion on the ferry in the framework of liminality see Sang and Huang, 2023). Gambling on the ferries is obviously not a regular or even weekly gambling activity for the Finnish population (Salonen et al., 2018).

“*Last year we were on the ferry to Tallinn with relatives, and as I remember, my grandmother put almost a euro in the slot machine. […] My grandmother won approx. 50 euros from this. Even though I didn't actually play more than a few spins, the event was truly an incredible experience due to the small odds. The day after the game, when we were in a restaurant in Tallinn, my grandmother spent a little more money there than usual, but otherwise the event was not celebrated as it was not such a huge amount. […] The experience has still remained much more memorable than the time we won just under 20 thousand euros as a result of my father's enthusiastic lottery playing.” (man, 25 years, most memorable gambling)*

The example narrative above illustrates very well the overall structure of the “ferry narratives.” The experience is remembered as an exceptional event and embedded in a familial/intimate contexture. The narrative also switches, at times, towards an economic logic in referring to the amounts gambled and won. Yet, primarily, this is a story about a beloved family member, who deserves the very unlikely (“small odds”) win (cf. Falk and Mäenpää, 1999). The importance of the familial/intimate aspect of gambling is highlighted in the last sentence. It may adhere towards an economic logic (the considerable win of nearly 20 thousand euros), but by pointing out that the grandmother's comparably minor win was more memorable, gambling becomes a matter of the familial and intimate realm.

## Discussion

In this article, I have shown that it is possible to discuss gambling without an actor-centred approach, instead using a systems theoretical lens to analyse gambling narratives. Overall, gambling is made communicable in economic and family/intimate terms. Identifying these frames allows developing four types of gambling: genuine monetary gambling, resonating monetary gambling, commensal gambling, and liminal gambling. Genuine monetary gambling is almost mono-contextural: gambling is the winning or losing of money, which is anticipated as self-evident. Resonating gambling shows richer poly-contextural structures: the winning or losing of money is embedded, but the economic logic takes prominence, resonating in other (mostly familial/intimate) contextures. Commensal gambling is bonding and binding. The rich poly-contextural networks are dominated by the familial/intimate contexture. Sharing the gambling experience is common in this type of gambling. Finally, the liminal type of gambling shows poly-contextural networks, with a predominance of the familial/intimate sphere. Similarly to the commensal type, gambling is bonding and binding, but here gambling and its context is transitory. Whereas, commensal gambling is sharing in rather close proximity, liminal gambling often spreads beyond the borders of the gambling activity, affecting wider areas of life.

Comparing the previously identified types of gamblers with the types of gambling developed in the present study shows that a shift away from gamblers, their background, their personality, and their motives to the gambling activity provides novel insights. Gambling can be a positive or negative experience. In many cases, it can be mundane if not indifferent. The findings of this study show that, in almost all instances, gambling is a matter of economic or familial/intimate logic. In the present framework of gambling harms, all harms have been treated as equal (Marionneau et al., 2023). Yet, the various kinds of harms are related to each other, where some forms of harm precede other harms and are the trigger for consecutive harm. These are the more interesting kinds of harms from the perspective of early prevention. Financial harm specifically has been discussed as one of the main and primary kinds of harm, being the cause of psychological distress and reduced well-being (Browne and Rockloff, 2018). The economic dimension has been also a core element in most of the narratives analysed in this study. The constant appearance of the familial/intimate dimension in the narratives indicate that, beside the financial harms, societal harms need to be treated as a category of harm in their own right and not as a consequence of personality disorders, psychological distress, or social deprivation.

The role of money in gambling is a topic of particular interest and constant debate. The main question tends to revolve around the issue of whether one is gambling with or for money and, consequently, if the gambler's relation to money is a predictor for problem gambling (e.g., Lee et al., 2007; Flack and Morris, 2015; Lloyd et al., 2021). Borch (2013: p. 84) considers money as a “*medium* [italics in original] of gambling, not what gambling actually is about.” Similarly, Kinnunen et al. (2016) found that, for casual gamblers, money is a tool to increase time spent gambling. Money has an effect on how gambling is experienced (ibid.). Money leaves its mark on what gambling is. In this study, I consider money instead as the symbolic generalised medium of communication of the economic system, which is particularly potent in making communication successful, and also more likely to be continued in other systemic logics (Luhmann, 1988). Money turns into a stake when it is gambled, and it then continues its journey further. A win is used to get drunk or to make bystanders happy; it also has the potential to make siblings jealous of each other. Lost money limits the gamblers' fiscal options, but it also limits their lives wherever the missing money has lost its possibility to resonate. The question on whether one is gambling for or with money thus becomes less pressing. Instead, tracing the money through various social systems and examining where and how the money resonates can inform us on how money is involved in creating a more positive or negative gambling experience and how money incites pleasure or gambling harm.

Money is central to gambling. Money as the generalised medium of the economic system is formidable in processing environmental complexity and contingency. This could explain why the monetary types of gambling appeared so ubiquitous. Yet, money is not only included in monetary types of gambling, but also in the familial/intimate types of gambling. This points to a considerable resonance of money in the familial system. The familial system is not a function system, and lacks a generalised medium of communication (Roth and Schütz, 2015). The contingency, innate in gambling, that is the uncertainty of winning or losing, is processed in the familial/intimate system by the logic of love–as everything in this system is. This puts the winning or losing of money in an additional, non-economic light and shows how gambling can cause societal harms directly, without the detour via the individual's economic hardships.

Interestingly, it seems that the most memorable gambling experiences tend to focus on the use of money, whereas the worst memories on the origin. This can be, on the one hand, interpreted as different meanings being attached to the money: for example, narrators regretted having wasted money they received as a present, while in other narratives money won becomes a good story to be told to friends (cf. Zelizer, 1997). On the other hand, money denotes a temporal dimension. Time is the essence of money; it does not satisfy present needs, but instead tames the uncertainty of the future (Esposito, 2010). Hence, while losing money reduces options and creates problems in the present, the more severe issue is that gambling losses deprive gamblers of possible futures. Lost possible futures are obviously hard if not impossible to measure, as one often does not know what such a future would have been like. Yet, from a comprehensive harm perspective, this is something to account for.

The narratives about the worst gambling experiences do not equal accounts of problem gambling. Yet, the reported problems, frustrations, and troubles are instances of minor gambling harm. These issues are insignificant on their own but when aggregated can have a significant impact on the population level (e.g., Browne et al., 2017). The aunt being angry “for weeks” due to her niece's lack of gambling expertise might be a rather mild form of gambling harm, but when suffered by many, this can become a matter of societal well-being. In addition, this example of the commensal type of gambling also displays how gambling can disrupt relations and thus burden well-being even independently of any money lost. Such a disruption of social relations also became obvious in instances where gambling as a gift (or gift money used for gambling) felt like a waste. While the, usually expected, reciprocity of gifting creates and upholds social relations, a “wasted” gift interrupts such continuous interchange and might even risk signalling to the donor directly that this relation is not wished for anymore.

Then again, gambling in a group has been identified as a factor decreasing gambling risks (Molde et al., 2017) and lonely gambling, on the other hand, indicates a problematic form of gambling (Egerer and Marionneau, 2015). What the approach of the present study and the developed types of gambling point to is, however, of a different character. Gambling harm can originate in society and social relationships, i.e., the harm happens in the social fabric itself. Studies looking at problem gamblers and the misery they cause to their close ones take the individual as the source of the harm. Furthermore, the majority of gamblers change their gambling intensity and have periods of heavier and reduced gambling (Reith and Dobbie, 2013). These alterations could be traced to changes in the gamblers' lives and their social context (ibid.). The findings of the present study demonstrate that gambling does not only change in intensity, but also in character. Whereas, Reith and Dobbie (2013) identified factors in gamblers' lives that were linked to changing gambling behaviour, the types of gambling identified in the current study offer an understanding of how various types of gambling affect gamblers' lives.

The sampling strategy limits the reach of the conclusions; there are probably other types of gambling to be discovered. Poker and sport betting narratives in particular were rare in this study's data. Future studies on the types of gambling need to address experiences of these games to a larger degree. It would also be intriguing to question if certain types of gambling correspond to particular games and, if so, which. The findings of the current study relate to and can be interpreted with numerous sociological approaches. In the limited space, many of these issues could be only touched upon, and in the future, these elaborations could be expanded. Yet, the results and their interpretation clearly show how fruitful it is to take a sociological approach to studying gambling. While the types of gambling presented here are “Finnish” types of gambling, the applied system theoretical analysis can serve gambling researchers beyond the Finnish case. Developing theoretically sound alternatives to the strongly individualised framework of gambling and particular problem gambling research is paramount in informing more useful and ethical gambling regulations.

A review on the available frameworks of conceptualising gambling harm (Marionneau et al., 2023) found that the number of frameworks is surprisingly limited but that even frameworks promoting a public health approach to gambling harms keep the individual as the pivot of gambling harm. While acknowledging and listing numerous societal harms, these can be traced towards the individual problem gambler. In order to facilitate the vital turn towards social, political, and commercial determinants of harm independent of the individual gambler, I suggested in this article a methodological shift away from actor-centric approaches towards a system theoretical approach focusing on societal processes, operations, and mechanisms.

## Data availability statement

The datasets presented in this article are not readily available as they can only be accessed by those involved in the project. Queries regarding the data should be directed to the author.

## Ethics statement

The studies involving human participants were reviewed and approved by Helsinki Ethical Review Board in Humanities and Social and Behavioural Sciences. Written informed consent for participation was not required for this study in accordance with the national legislation and the institutional requirements.

## Author contributions

ME wrote the manuscript on his own.

## Appendix

### Instructions to participants

Write a story about an ordinary gambling experience of yours! (Describe for example: With whom did you gamble? When and where did you gamble? How did it feel? What happened before and after you gambled?)

[Kirjoita tarina tavallisesta rahapelikokemuksestasi! (kuvaile esimerkiksi: Kenen kanssa pelasit? Milloin ja missä pelasit? Miltä se tuntui? Mitä tapahtui ennen pelaamista ja mitä sen jälkeen?)]

Write a story about your most memorable gambling experience! (Describe for example: With whom did you gamble? When and where did you gamble? How did it feel? What happened before and after you gambled?)

[Kirjoita tarina ikimuistoisesta rahapelikokemuksestasi! (kuvaile esimerkiksi: Kenen kanssa pelasit? Milloin ja missä pelasit? Miltä se tuntui? Mitä tapahtui ennen pelaamista ja mitä sen jälkeen?)]

Write a story about your worst gambling memory! (Describe for example: With whom did you gamble? When and where did you gamble? How did it feel? What happened before and after you gambled?)

[Kirjoita tarinan surkeimmasta rahapelimuistostasi! (kuvaile esimerkiksi: Kenen kanssa pelasit? Milloin ja missä pelasit? Miltä se tuntui? Mitä tapahtui ennen pelaamista ja mitä sen jälkeen?)]
